# Circ_0051428 targeting miR-885-3p/MMP2 axis enhances the malignancy of cervical cancer

**DOI:** 10.1515/med-2023-0858

**Published:** 2024-03-13

**Authors:** Caixian Song, Liping Chen

**Affiliations:** Department of Gynecology and Obstetrics, Wuhan Fourth Hospital, Wuhan 430030, Hubei, China; Department of Gynecology and Obstetrics, Wuhan Fourth Hospital, No. 76 Jiefang Avenue, Qiaokou District, Wuhan 430030, Hubei, China

**Keywords:** cervical cancer, circ_0051428, miR-885-3p, MMP2, proliferation, invasion

## Abstract

Circular RNAs (circRNAs) are key regulators of cervical cancer (CC) progression. This study aimed to elucidate the role and mechanism of circ_0051428, a novel circRNA, in CC tumorigenesis. Quantitative real-time polymerase chain reaction and western blotting analyses confirmed that circ_0051428 and matrix metalloprotein-2 (MMP2) were overexpressed in CC, whereas the microRNA miR-885-3p was poorly expressed. After performing a series of *in vitro* and *in vivo* experiments, circ_0051428 knockdown was shown to repress CC cell invasion and proliferation *in vitro*, and hamper tumor formation *in vivo*. Dual-luciferase reporter and RNA-binding protein immunoprecipitation experiments verified that circ_0051428 interacts with miR-885-3p to regulate the target gene MMP2 of miR-885-3p in CC. In addition, miR-885-3p knockdown offset the anticancer effects of circ_0051428 or MMP2 knockdown on CC cell malignancy. Overall, this study revealed that circ_0051428 executes a tumor-promoting function in CC pathogenesis by modulating the miR-885-3p/MMP2 axis. Our findings provide a novel approach for CC treatment.

## 1 Introduction

Cervical cancer (CC), a prevalent gynecological malignancy, ranks as the fourth leading cause of tumor-related mortality in females [1,2]. The main causes of CC tumorigenesis are human papillomavirus infection and epigenetic, immunological, and environmental factors [3]. With the development of therapeutic strategies, including hysterectomy, chemotherapy, and radiotherapy, the 5-year survival rate for patients with early stage CC is above 90% [4–6]. However, approximately 67% of patients with CC are diagnosed with terminal CC and have an extremely poor 5-year survival rate (less than 17%) due to frequent recurrence and high metastasis rates that render standard treatment strategies ineffective [7,8]. Therefore, exploring the mechanism of metastasis underlying the pathogenesis of CC may provide new clues that aid in the development of better therapeutic strategies for patients with CC.

Circular RNA (circRNAs) are a novel class of non-coding RNA (ncRNA) molecules in eukaryotes that exhibit a unique circular-closed ring structure with more stable covalent bonds than linear mRNA [9–12]. Recently, numerous studies have revealed the regulatory functions of circRNAs in CC progression; for example, circ_0000388 is overexpressed in CC and has been identified as an effective marker in patients with CC [13]. CC patients with lymphatic metastasis show relatively higher circ_0000745 expressions than those with localized CC [14]. Overexpression of both circ_0003221 and circ_0084927 has been detected in CC tissues, and knockdown of these two circRNAs inhibits proliferation and metastasis, while accelerating apoptosis in CC [15,16]. circ_0051428, also known as hsa_circ_102566, is a novel circRNA found in human cancers. According to previous reports, circ_0051428 is an oncogenic circRNA involved in thyroid cancer [17]. Moreover, circ_0051428 has been found to be upregulated in hepatocellular carcinoma tissues [18]. These findings indicate that dysregulation of circ_0051428 may participate in tumorigenesis, although there is still no relevant research on its role in CC progression. Therefore, this study aimed to explore the role of circ_0051428 in CC.

MicroRNAs (miRNAs) are 20–23 nucleotides in length and belong to the ncRNA family [19]. The regulatory functions of miRNAs in CC tumorigenesis have been extensively studied. For instance, the expressions of miR-9-5p and miR-92a are remarkably higher in CC tissues than in negative tissues, and are closely linked to the hyperproliferation and rapid metastasis of CC cells [20,21]. A decline in miR-195-3p, miR-218, and miR-362 expressions has been found in CC tissues, and these miRNAs exert cancer-inhibiting functions by suppressing cell metastasis and growth in CC [22–24]. These studies revealed that different miRNAs play different roles in CC. Additionally, miRNAs have been shown to bind to circRNAs, thereby regulating CC tumorigenesis [25–27]. Furthermore, it has been found that miR-885-3p could bind to circ_0051428 and that miR-885-3p exerts antitumor effects in glioblastoma [28], colon cancer [29], and gastric cancer [30]. Moreover, both the function of miR-885-3p and whether circ_0051428 can modulate it in the progression of CC have not yet been revealed.

Given the key regulatory role of circRNAs in CC, this study aimed to explore the expression pattern and mechanism of action of circ_0051428 in CC. The findings of this study provide a prospective target for the clinical treatment of CC.

## 2 Methods

### 2.1 Specimen tissue collection

Patients with CC (*n* = 38) were registered in our hospital from August 2020 to October 2021. None of the patients received any treatment before enrollment or underwent histological examination for diagnosis. The tumor and corresponding normal tissues were surgically obtained. The samples had been kept at −80°C until they were needed for the experiments.

### 2.2 Cell culture

Four CC cell lines, HeLa (cat. #BNCC342189), CaSki (cat. #BNCC354385), SiHa (cat. #BNCC337881), and C33A (cat. #BNCC354329), were obtained from the Bena Culture Collection (Beijing, China). HcerEpic (cat. #GPC0088), a normal human cervical epithelial cell line, was obtained from the China Center for Type Culture Collection (Wuhan, China). The cell lines were inoculated in Dulbecco’s Modification of Eagle’s Medium (DMEM) with 10% fetal bovine serum (FBS), both from Invitrogen (Carlsbad, CA, USA), and then maintained at 37°C in an incubator containing 5% CO_2_. All cells that achieved the logarithmic growth phase were used for subsequent experiments.

### 2.3 Cell transfection

Two small interfering RNAs targeting circ_0051428 (si-circ-1 and si-circ-2), the matrix metalloprotein-2 (MMP2) gene (si-MMP2-1 and si-MMP2-2), miR-885-3p mimic, and miR-885-3p inhibitor (inhibitor) as well as their negative controls (si-NC, miR-NC, and inhibitor-NC) were sourced from Sangon Biotech (Shanghai, China). Lipofectamine 3000 (Invitrogen) was used to introduce the aforementioned agents into HeLa and CaSki cells. Two days later, the transfected HeLa and CaSki cells were collected for functional experiments.

### 2.4 Quantitative real time polymerase chain reaction (qPCR)

Total RNA was isolated from CC cells and tissues using an RNA extraction kit (Cwbio, Beijing, China). After reverse transcription to cDNA using the Evo M-MLV reverse transcription reagent (Accyrate Biology, Changsha, China), qRT-PCR was performed using the SYBR Green Kit (Accyrate Biology) on an ABI 7900 Real-Time PCR System (Applied Biosystems, Foster City, CA, USA). U6 and glyceraldehyde-3-phosphate dehydrogenase (GAPDH) were used for normalization of miR-885-3p and circ_0051428 and MMP2, respectively. The 2^−ΔΔCt^ approach was adopted to determine the expression levels of miR-885-3p, MMP2, and circ_0051428. Primers used are listed in Table 1.

**Table 1:** Real-time PCR primer synthesis list

Gene	Sequences	
circ_0051428	Forward	5′-ATCTGCTTCCAGGCCTCATA-3′
	Reverse	5′-AGGCAGTCACCTCCACCTC-3′
miR-885-3p	Forward	5′-CGTTAGGCAGCGGGGTGTAG-3′
	Reverse	5′-ATCCAGTGCAGGGTCCGAGG-3′
MMP2	Forward	5′-GCATCCAGACTTCCTCAGGC-3′
	Reverse	5′-ATTAGCGCCTCCATCGTAGC-3′
U6	Forward	5′-CTCGCTTCGGCAGCACA-3′
	Reverse	5′-AACGCTTCACGAATTTGCGT-3′
GAPDH	Forward	5′-AGAAAAACCTGCCAAATATGATGAC-3′
	Reverse	5′-TGGGTGTCGCTGTTGAAGTC-3′

### 2.5 RNase R treatment

RNase R (3 U/μg; Geneseed Biotech, Guangzhou, China) was used to treat the extracted RNA for 1 h at 37°C. The circ_0051428 and linear gene RELB expression levels were determined by qRT-PCR analysis, as described above.

### 2.6 Subcellular location assay

A Cytoplasmic/Nuclear Extraction Kit (Thermo Fisher Scientific, Waltham, MA, USA) was used to segregate the nuclear and cytoplasmic fragments of CaSki and HeLa cells and to isolate their RNAs. RNA expressions were then quantified using qRT-PCR. The nuclear and cytoplasmic controls were U6 and GAPDH, respectively.

### 2.7 RNA immunoprecipitation assay (RIP)

RIP was performed using the Magna RIP RNA-Binding Protein Immunoprecipitation Kit (Millipore, Bedford, MA, USA). HeLa and CaSki cells were treated with RIP lysis buffer, conjugated with magnetic beads, and incubated with anti-Ago2/anti-IgG (1:3,000; Millipore). qRT-PCR was conducted to assess the RNA enrichment.

### 2.8 Dual luciferase reporter (DLR) assay

CircInteractome was used to predict the circ_0051428–miR-885-3p binding site, and TargetScan was used to predict the target relationship of MMP2 with miR-885-3p. The sequences of circ_0051428 and MMP2 containing miR-885-3p binding sites were merged to pGL3 (Promega, Madison, WI, USA) to develop the wild-types (WTs), namely circ_0051428 WT and MMP2 WT. Thereafter, the miR-885-3p binding sites were separately mutated into circ_0051428 and MMP2 fragments before being ligated into a pGL3 vector to construct the mutants (MUTs), namely circ_0051428-MUT and MMP2-MUT. Lipofectamine 3000 was used to transfect HeLa and CaSki cells with a combination of the constructed vector and miR-885-3p mimic/NC. Relative luciferase activity was measured using a Dual-Glo Luciferase assay system (Promega).

### 2.9 Cell viability assay

HeLa and CaSki cells were seeded into 96-well plates (4 × 10^3^ cells/well) and cultured for a specified duration (0, 24, 48, or 72 h). Thereafter, Cell Counting Kit-8 (CCK8; 15 μL, Sangon Biotech) was supplied to each well and the cells were incubated for two more hours. The optical density was measured at 450 nm using a microplate reader (DR-3518G; Hiwell Diatek, Wuxi, China).

### 2.10 Cell colony assay

CaSki and HeLa cells were inoculated into 6-well plates (1 × 10^3^ cells/well) and incubated for approximately 2 weeks. Afterward, the cells were rinsed, immobilized, stained, and then sustained at 25°C. The cell colonies (>50 cells) were visualized using an Olympus light microscope (Tokyo, Japan).

### 2.11 Transwell invasion assay

The upper chambers were pre-coated using Matrigel from BD Biosciences (San Jose, CA, USA). HeLa and CaSki cells (2 × 10^5^ cells for each well) were cultured in the top chambers that contained serum-free DMEM (500 µL). Additionally, the lower chamber was filled with DMEM (750 µL) that contained 10% FBS. Approximately 24 h later, the lower chamber cells were fixed with methanol for 15 min before staining with 0.1% crystal violet for 30 min. Cells were counted under a light microscope (Olympus).

### 2.12 Western blotting

HeLa and CaSki cells were dissolved in RIPA lysis buffer (BOSTER, Wuhan, China) for total protein extraction. Thereafter, their concentrations were quantified using the Rapid Gold BCA Kit (Pierce, Rockford, IL, USA). Sodium dodecyl sulfate polyacrylamide gel electrophoresis (10%) was used to electrophorese a total of 30 µg of protein before they were moved to polyvinylidene fluoride membranes. The membranes were then sealed by being treated for 1 h with 5% nonfat milk at room temperature. Afterward, primary antibodies, namely MMP2 (Abcam, Cambridge, UK; 1:1,000) and GAPDH (Abcam; 1:1,000), were added and maintained overnight at 4°C. Thereafter, they were further incubated for 30 min at 25°C with a specific secondary antibody (Abcam; 1:3,000). GAPDH was used as the internal control. An enhanced chemiluminescence detection kit (Tanon, Shanghai, China) and Gel-Pro Analyzer 4.0 (Media Cybernetics, Silver Spring, MD, USA) were used to visualize and analyze the bands.

### 2.13 Mouse xenograft model

Five-week-old BALB/c nude mice (22–28 g) obtained from Esebio (Shanghai, China) were randomly assigned to the sh-circ/NC group (*n* = 5 mice per group). Sh-circ and sh-NC were first integrated individually into lentivirus vectors prior to their delivery into the HeLa cells (3 × 10^5^ cells/100 μL). Subsequently, the transfected cells (3 × 10^5^ cells/100 μL) were administered subcutaneously into each mouse. Tumor length (*A*) and width (*B*) were documented weekly to compute tumor volume using the formula *V* = 1/2 × *AB* ^2^. After 5 weeks, the mice were euthanized with a pentobarbital sodium overdose. Thereafter, tumors were collected and weighed. This study was approved by the Animal Ethics Committee of our hospital.

### 2.14 Statistical analyses

SPSS 23.0 software (Chicago, IL, USA) was used for analyses, and the data were presented as the mean ± SD. The student’s *t*-test was used to test the differences between groups. Furthermore, two-way analysis of variance (ANOVA) with Sidak’s multiple comparisons test or one-way ANOVA plus Tukey’s *post*-*hoc* test was adopted among more than two groups. The relationships between miR-885-3p and circ_0051428 or MMP2 were determined using Pearson’s correlation coefficients. Differences were considered significant at *P* < 0.05.


**Ethical statement:** The Ethics Committee of Wuhan Fourth Hospital (Wuhan, China) approved the present study. The processing of clinical tissue samples had been in strict compliance with the ethical standards of the Declaration of Helsinki. All patients signed a written informed consent. This animal experiment was conducted in accordance with the ARRIVE guidelines and was authorized by the Animal Ethics Committee of Wuhan Fourth Hospital.
**Consent to participate:** All patients signed a written informed consent.

## 3 Results

### 3.1 circ_0051428 is overexpressed in CC

**Figure 1:**
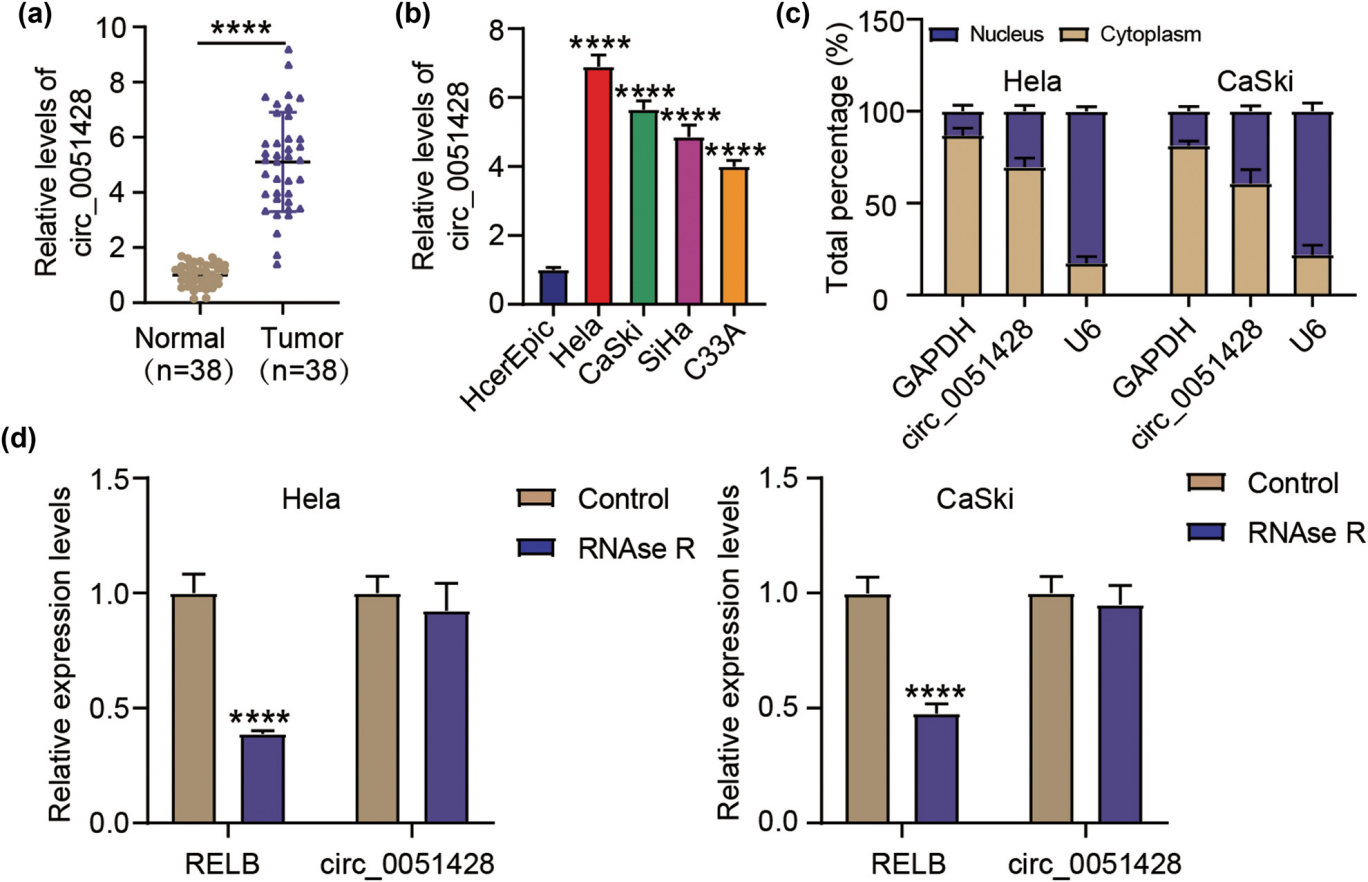
Circ_0051428 is overexpressed in cervical cancer. (a) The circ_0051428 expressions in normal and CC tissues were estimated via qRT-PCR. *P* < 0.0001 vs Normal. (b) The circ_0051428 levels in the CC cell lines (CaSki, C33A, HeLa, and SiHa) as well as in the HcerEpic cells were quantified via qRT-PCR. ^**^ *P* < 0.01 vs HcerEpic. (c) Circ_0051428 levels within the HeLa and CaSki nuclei and cytoplasm. (d) The circ_0051428 and linear RELB expressions in total cellular RNA incubated with RNase R. ^**^ *P* < 0.01 vs Control.

circ_0051428 expression was detected in 38 pairs of CC and normal tissues collected in this study. As illustrated in Figure 1a, circ_0051428 was more highly expressed in tumors than in the matched normal tissues. Interestingly, circ_0051428 was also highly expressed in CC cell lines (CaSki, C33A, HeLa, and SiHa), but poorly expressed in HcerEpic cells (Figure 1b). Because their circ_0051428 expressions were relatively high, both HeLa and CaSki cells were selected for subsequent cellular experiments. The results of the subcellular localization experiment indicated that circ_0051428 was predominantly located within the cytoplasm, with a cytoplasm/nucleus distribution ratio of approximately 70%/30% (Figure 1c). Furthermore, exposure to RNase R was found to have no notable effects on circ_0051428 expression but remarkably diminished the expression of linear RELB (Figure 1d). These results suggest that circ_0051428 has a stable structure and is overexpressed in CC cells.

### 3.2 Silencing of circ_0051428 represses the invasion and proliferation of cells *in vitro* as well as tumor xenograft growth *in vivo*

**Figure 2:**
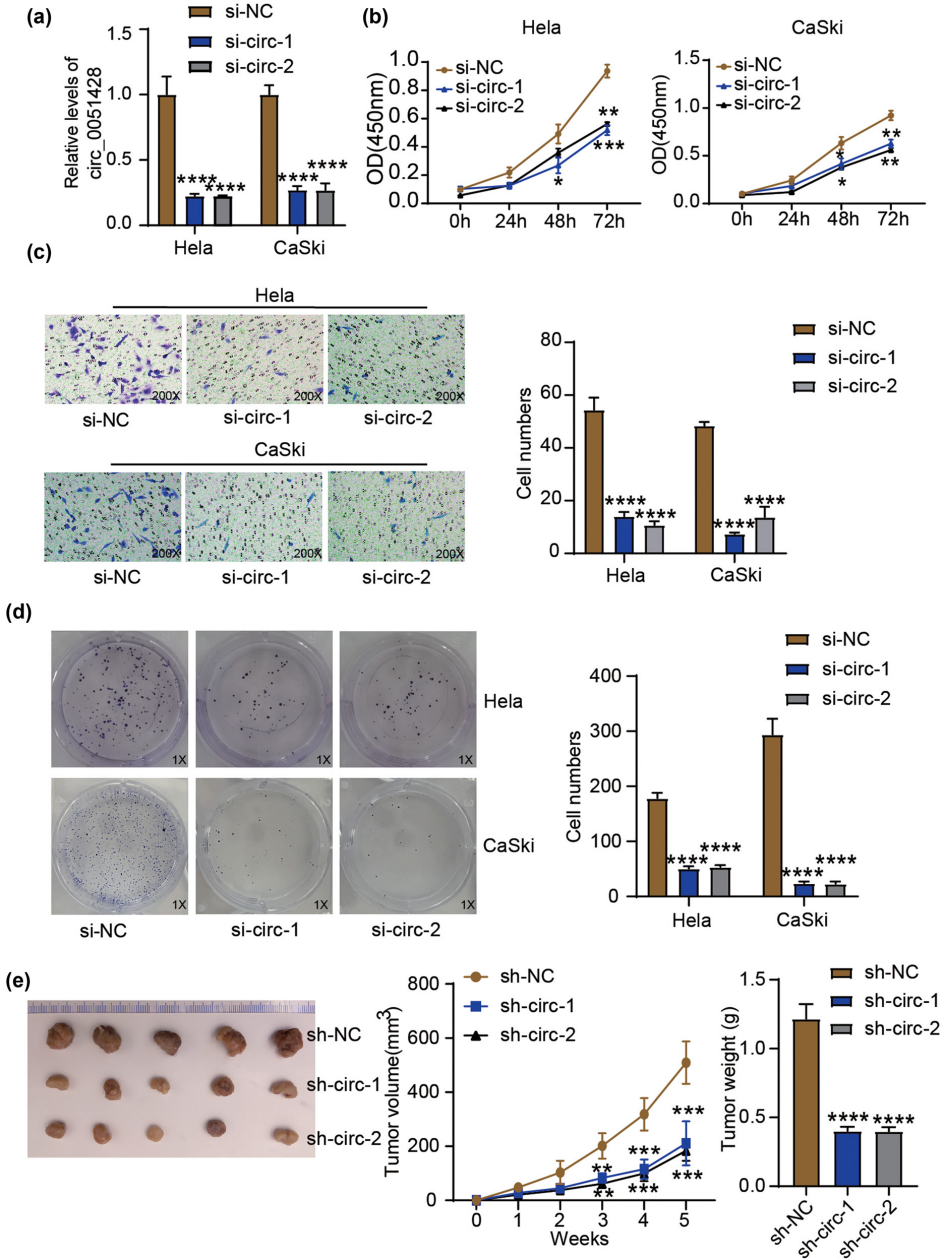
Silencing circ_0051428 represses the invasion and proliferation of cervical cancer cells *in vitro* as well as the growth of tumor xenograft *in vivo*. (a) The circ_0051428 expression in CaSki and HeLa cells, following si-circ-1, si-circ-2, or si-NC transfection, was estimated via qRT-PCR. ^**^ *P* < 0.01 vs si-NC. (b) HeLa and CaSki cells carrying si-circ-1, si-circ-2, or si-NC were evaluated for viability by means of the CCK-8 experiment. ^**^ *P* < 0.01 vs si-NC. (c) The number of invasive HeLa and CaSki cells following their transfection with si-circ-1, si-circ-2, or si-NC was assessed in the Transwell invasion experiment. ^**^ *P* < 0.01 vs si-NC. (d) The number of HeLa and CaSki cell colonies following their transfection with si-circ-1, si-circ-2, or si-NC was tallied in the colony formation experiment. ^**^ *P* < 0.01 vs si-NC. (e) Pictures, weights, and volumes of xenograft that resulted from injecting each mouse with HeLa cells carrying sh-circ-1, sh-circ-2, or sh-NC. The tumor volume was documented weekly. The tumor weight was measured after 5 weeks. ^**^ *P* < 0.01 vs sh-NC.

The expression of circ_0051428 decreased in HeLa and CaSki cells following transfection with si-circ-1 and si-circ-2 (Figure 2a). This indicates that si-circ was successfully transfected into HeLa and CaSki cells to knockdown circ_0051428. The viability of CaSki and HeLa cells was significantly reduced by circ_0051428 knockdown (Figure 2b). circ_0051428 deficiency also suppressed the invasive abilities of HeLa and CaSki cells (Figure 2c). Furthermore, a dramatic decline was found in the number of cell colonies in the two si-circ groups compared to that in the si-NC group (Figure 2d). The effect of circ_0051428 deficiency on xenograft tumor growth was also investigated. As depicted in Figure 2e, both the volume and weight of the tumor xenografts in the two sh-circ groups were notably lower than those in the tumors from the sh-NC group. These results indicate that circ_0051428 silencing has an antitumor role both *in vitro* and *in vivo*.

### 3.3 Circ_0051428 targets miR-885-3p

**Figure 3:**
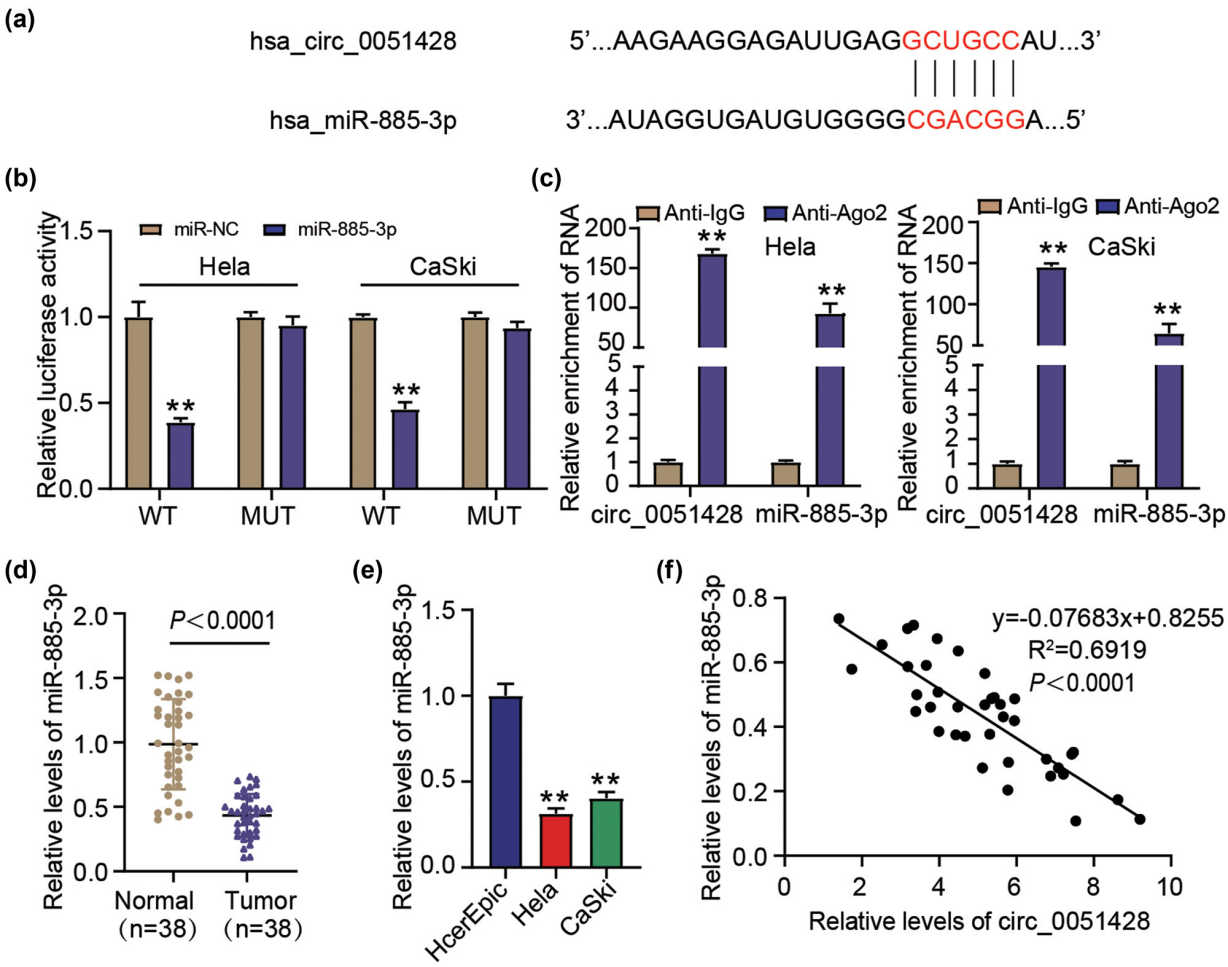
Circ_0051428 targets miR-885-3p. (a) CircInteractome predicted the binding site between circ_0051428 and miR-885-3p. (b) Luciferase activities in HeLa and CaSki that have a combination of pGL3-circ_0051428 WT/MUT and miR-885-3p mimic/NC had been assessed via the dual luciferase experiment. ^**^ *P* < 0.01 vs miR-NC. (c) Circ_0051428’s interaction with miR-885-3p was verified using the results of the RIP experiment. ^**^ *P* < 0.01 vs anti-IgG. (d) The miR-885-3p levels in normal and CC tissues were estimated via qRT-PCR. *P* < 0.0001 vs Normal. (e) The miR-885-3p levels among the CC cell lines (HeLa and CaSki) as well as in HcerEpic cells were gauged by conducting qRT-PCR. **P < 0.01 vs HcerEpic. (f) In CC tissues, the correlation of circ_0051428 expression with that of miR-885-3p had been ascertained using Pearson’s correlation coefficient.

Circlnteractome (https://circinteractome.nia.nih.gov/index.html) was used to predict the latent circ_0051428–miR-885-3p binding site (Figure 3a). In view of the outcomes of the DLR assay, the introduction of the miR-885-3p mimic into HeLa and CaSki cells dramatically diminished the luciferase activity of circ_0051428 WT, but had no apparent influence on that of circ_0051428 MUT (Figure 3b). The RIP experiment further revealed that relative to the anti-IgG group, circ_0051428 and miR-885-3p were more enriched in the anti-Ago2 group (Figure 3c). miR-885-3p expressions in tumor tissues and CC cells were explored. As shown in Figure 3d and e, relative to the respective controls, miR-885-3p was poorly expressed in CC tumors and cell lines. Moreover, an inverse correlation was observed between circ_0051428 and miR-885-3p expression in CC tissues (Figure 3f). Therefore, it can be inferred that circ_0051428 negatively regulates miR-885-3p expression in CC.

### 3.4 mir-885-3p suppression partly abates the anti-tumor influences of circ_0051428 knockdown on the invasion and proliferation of CC cells

**Figure 4:**
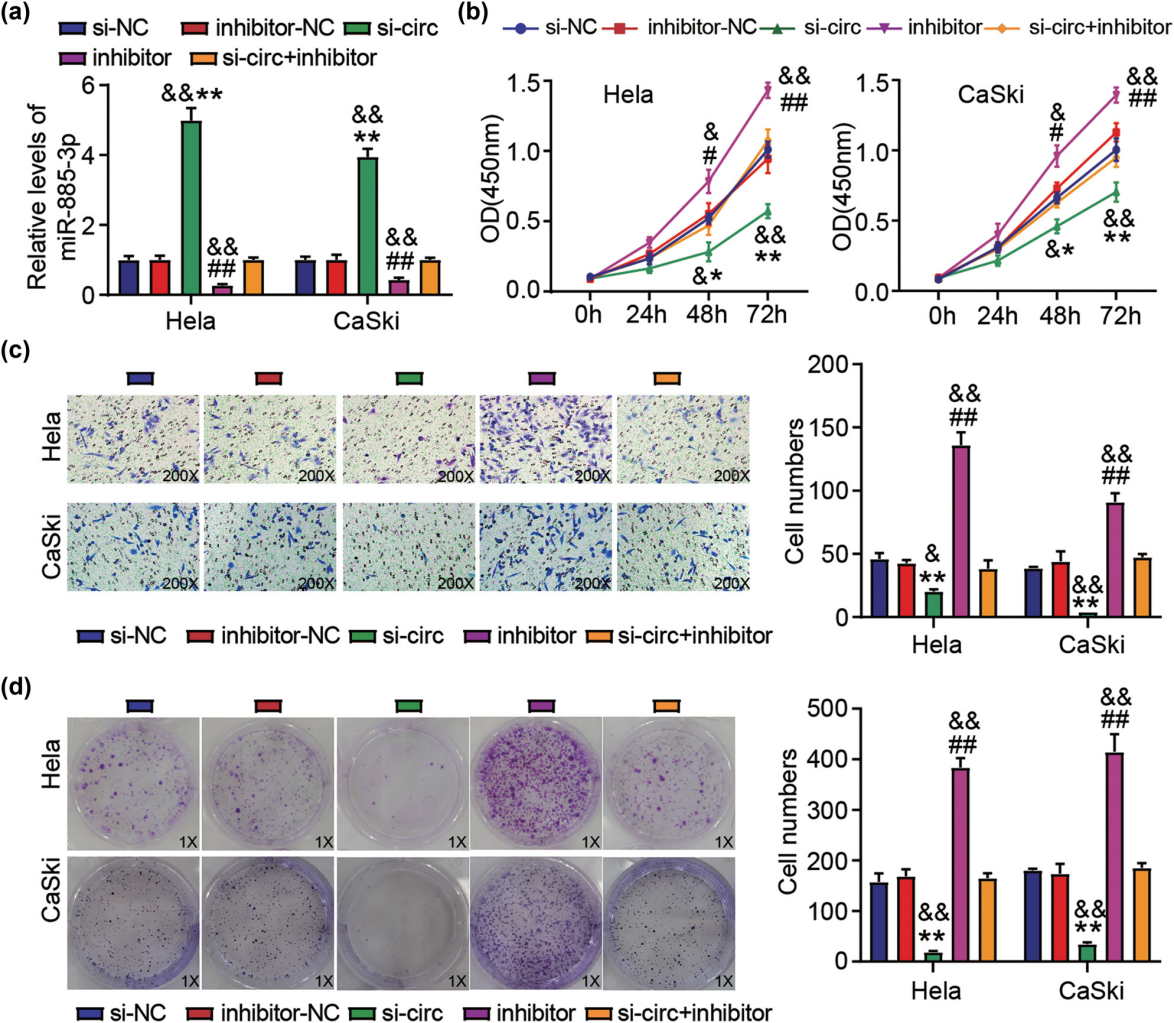
mir-885-3p inhibition partly abates the anti-tumor effect of circ_0051428 knockdown on cervical cancer cell invasion and proliferation. Si-circ, miR-885-3p inhibitor (inhibitor), inhibitor-NC, si-NC, and si-circ + inhibitor were introduced into HeLa and CaSki cells. (a) The miR-885-3p levels in transfected HeLa and CaSki cells were estimated via qRT-PCR. (b) The transfected CaSki and HeLa cells were tested for viability via the CCK-8 experiment. (c) The number of transfected invasive cells was determined in the Transwell invasion experiment. (d) The number of transfected HeLa and CaSki cell colonies was determined by executing the colony formation experiment. ^*^ *P* < 0.05, ^**^ *P* < 0.01 vs si-NC. ^#^ *P* < 0.05, ^##^ *P* < 0.01 vs inhibitor-NC. ^&^ *P* < 0.05, ^&&^ *P* < 0.01 vs si-circ + inhibitor.

To probe the interaction of miR-885-3p with circ_0051428 in CC progression *in vitro*, an miR-885-3p inhibitor was delivered into HeLa and CaSki cells. It was observed that circ_0051428 silencing enhanced miR-885-3p expression; however, this enhancement was suppressed by inhibitor transfection (Figure 4a). More importantly, transfection with the miR-885-3p inhibitor partly eliminated the stimulatory effect of circ_0051428 deficiency on miR-885-3p levels in HeLa and CaSki cells (Figure 4a). Functionally, inhibition of miR-885-3p exerted positive effects on the viability (Figure 4b), invasion (Figure 4c), and colony number (Figure 4d) of HeLa and CaSki cells. As expected, the diminished CC cell invasion and proliferation caused by circ_0051428 knockdown were offset by miR-885-3p inhibitor transfection in HeLa and CaSki cells (Figure 4b–d). These findings further validated the interaction between circ_0051428 and miR-885-3p in CC tumorigenesis.

### 3.5 mir-885-3p targets MMP2

**Figure 5:**
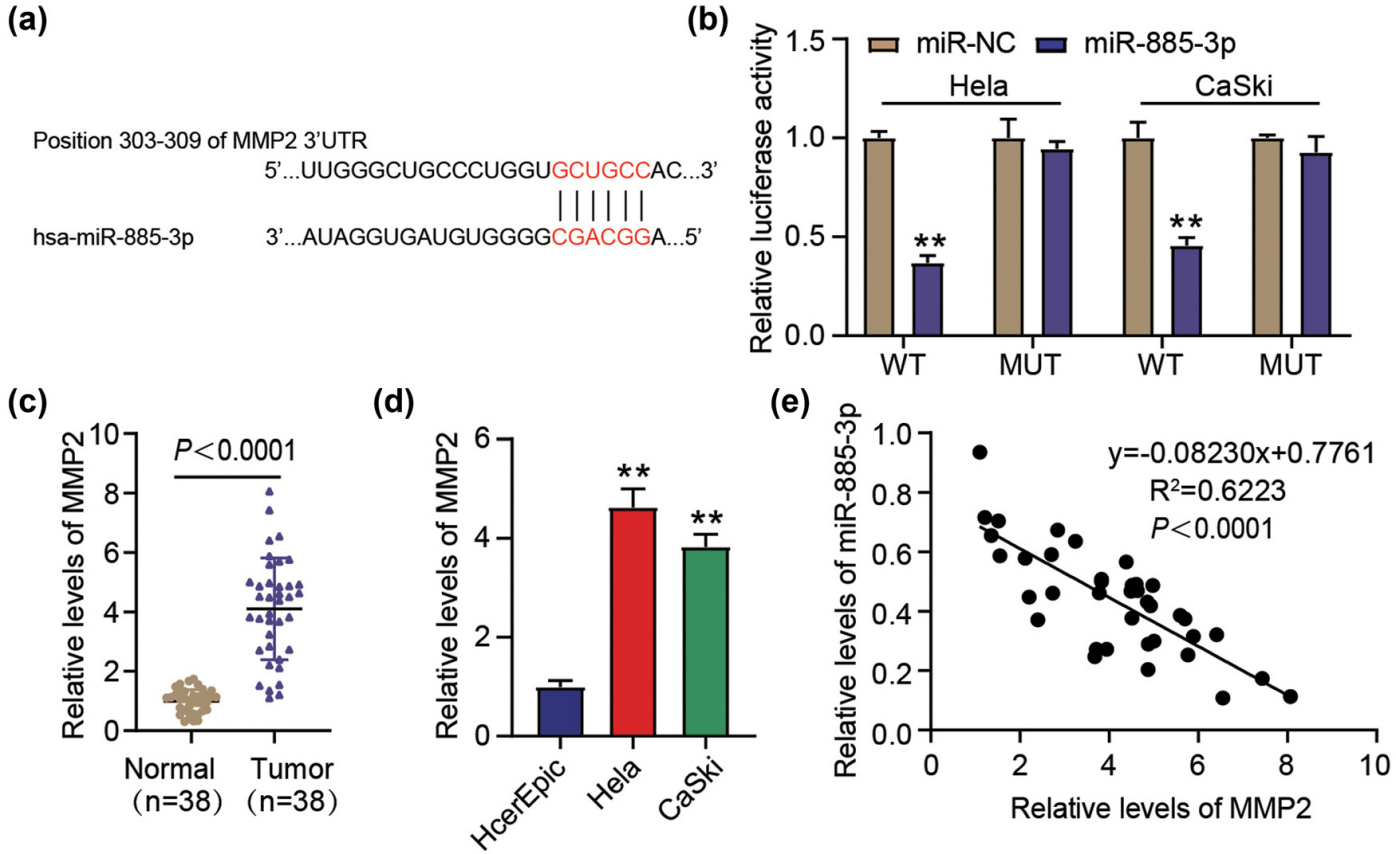
mir-885-3p targets MMP2. (a) TargetScan predicted the putative sites between miR-885-3p and MMP2. (b) The target relationship of MMP2 with miR-885-3p was substantiated by the outcomes of the dual luciferase experiment. ^**^ *P* < 0.01 vs miR-NC. (c) The amount of MMP2 in CC and normal tissues was estimated via qRT-PCR. *P* < 0.0001 vs Normal. (d) The MMP2 expression in HcerEpic and CC (HeLa and CaSki) cells was quantified via qRT-PCR. ^**^ *P* < 0.01 vs HcerEpic. (e) The association of miR-885-3p expression in CC tissues with that of MMP2 was ascertained using Pearson’s correlation coefficient.

The miR-885-3p–MMP2 binding site was predicted using TargetScan (https://www.targetscan.org/vert_80/) (Figure 5a). The results of the DLR experiment showed that the luciferase activity of the MMP2 WT/miR-885-3p mimic group declined considerably compared to that of the MMP2 MUT/miR-NC group. Interestingly, the MMP2 MUT/miR-885-3p mimic group did not exhibit statistically significant changes in luciferase activity (Figure 5b). Elevated levels of MMP2 expression were observed in CC tumors and cell lines compared to those in normal tissues and cells, respectively (Figure 5c and d). Furthermore, miR-885-3p expression in CC tumors was inversely associated with MMP2 expression (Figure 5e). Taken together, these results indicate that miR-885-3p regulates MMP2, and MMP2 is a miR-885-3p downstream gene.

### 3.6 mir-885-3p interacts with MMP2 to influence CC cell progression *in vitro*

**Figure 6:**
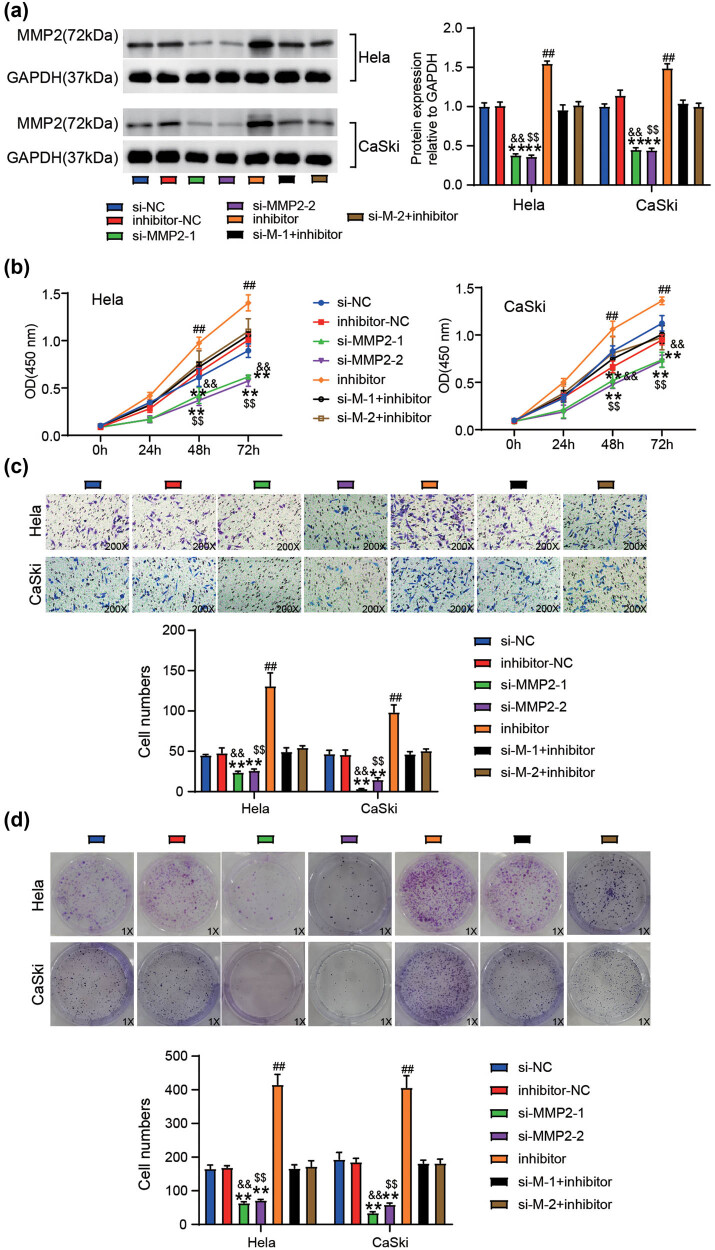
mir-885-3p interacts with MMP2 to influence cervical cancer progression *in vitro*. si-MMP2-1, si-MMP2-2, inhibitor, inhibitor-NC, si-NC, si-MMP2-1 + inhibitor, and si-MMP2-2 + inhibitor were transfected into CaSki and HeLa cells. (a) The levels of MMP2 protein in the transfected HeLa and CaSki were estimated via western blotting. (b) The viabilities of the transfected CaSki and HeLa were evaluated via the CCK-8 experiment. (c) The number of transfected invasive cells was assessed via the Transwell invasion experiment. (d) The number of transfected CaSki and HeLa cell colonies was assessed via the colony formation experiment. ^**^ *P* < 0.01 vs si-NC. ^##^ *P* < 0.01 vs inhibitor-NC. ^&&^ *P* < 0.01 vs si-MMP2-1 (si-M-1) + inhibitor. ^$$^ *P* < 0.01 vs si-MMP2-2 (si-M-2) + inhibitor.

MMP2 protein levels were determined after CaSki and HeLa cells were transfected with si-NC, inhibitor-NC, si-MMP2-1, si-MMP2-2, miR-885-3p inhibitor, si-MMP2-1 + miR-885-3p inhibitor, or si-MMP2-2 + miR-885-3p inhibitor. As shown in Figure 6a, MMP2 protein levels decreased after si-MMP2 transfection but increased after transfection with the miR-885-3p inhibitor. Downregulation of MMP2 markedly attenuated the stimulatory effect of miR-885-3p downregulation on MMP2 protein expression. The mechanism of action of the miR-885-3p/MMP2 regulatory axis in the progression of CC cells *in vitro* was also evaluated. As expected, silencing MMP2 produced inhibitory effects on CC cell viability (Figure 6b), invasion (Figure 6c), and colony formation (Figure 6d). Furthermore, as illustrated in Figure 6b–d, silencing MMP2 with two siRNAs offset the cancer-promoting role of miR-885-3p inhibition in the invasion and proliferation of the cells. This suggested that miR-885-3p interacts with MMP2 to regulate CC progression.

## 4 Discussion

CC is a malignant gynecological tumor with a high rate of metastasis and recurrence rate [31]. Several circRNAs have been reported to participate in the progression of CC [32–34]. Herein, it was found that circ_0051428 was upregulated in CC and that silencing of circ_0051428 inhibited proliferation, invasion, and tumor growth. Moreover, this study also proved that miR-885-3p knockdown could reverse the anticancer effects of circ_0051428 silencing on CC cell malignancy, because circ_0051428 interacts with miR-885-3p to regulate the target gene MMP2.

Emerging evidence has shown high expression levels of circRNAs, such as circ_0000515 [25], circ_0102171 [26], and circ_0000263 [27], in CC, where they play an oncogenic role in tumorigenesis. Similar to the results of previous studies, we observed that circ_0051428 was overexpressed in CC tumors and cell lines. Hence, we speculate that circ_0051428 may also function as an oncogenic factor in CC. Recent studies have shown that overexpressed circRNAs can serve as key regulators of CC tumorigenesis by affecting various cellular processes. For instance, Xie et al. demonstrated that circ_0003221 interference dramatically hinders the development of CC by hampering epithelial–mesenchymal transition, a key process in cancer metastasis [15]. Meng et al. reported that circ_0000388 deletion considerably limits the proliferative and migratory capacities of CC cells [13]. Jiao et al. discovered that circ_0000745 deficiency decreases cell viability and metastasis in CC [14]. Here, we observed that silencing circ_0051428 resulted in reduced proliferation and invasion of CC cells *in vitro*. The inhibitory effects of circ_0051428 on tumor formation were further validated *in vivo*. Hence, circ_0051428 silencing may impede CC development by controlling cancer cell growth and infiltration.

mir-885-3p generally exerts cancer-inhibiting functions in human cancers, such as glioblastoma [28], colon cancer [29], and gastric cancer [30]. In the tumorigenesis of these cancers, miR-885-3p expression was found to decrease, thereby affecting cancer progression. Similarly, low miR-885-3p levels have been detected in CC cells and tissues. Our experimental data showed that miR-885-3p was beneficial for CC tumorigenesis. It has been reported that circRNAs generally act on miR-885-3p sponges to mediate tumorigenesis, such as circ_SFMBT2-miR-885-3p in gastric cancer [35] and circ_TUBGCP3-miR-885-3p in lung adenocarcinoma [36]. We speculated that there may also be interactions between circ_0051428 and miR-885-3p during CC progression. As expected, miR-885-3p was identified as a downstream target of circ_0051428 in CC. Rescue assays further demonstrated that miR-885-3p downregulation counteracted the repressive effect of circ_0051428 silencing on the invasion and proliferation of CC cells. Therefore, we concluded that silencing circ_0051428 represses CC development via the regulation of miR-885-3p.

The deregulation of MMPs during malignant transformation and the resulting disruption of the extracellular matrix (ECM) of normal tissues are crucial for tumor metastasis [37]. MMP2, a 72 kDa zinc-dependent protein belonging to the MMPs family, cleaves ECM components and plays a key role in tumor growth and metastasis [38,39]. Increased MMP2 levels have been found to trigger the proliferation and metastasis of breast [40], ovarian [41], and lung [42] cancers. In this study, MMP2 overexpression was detected in CC cells and tissues. Furthermore, MMP2 deficiency was confirmed to repress the invasion and proliferation of CC. Similar to our results, several previous studies have reported elevated MMP2 expression in CC, which is linked to cell migration and invasion [43,44]. These data, to some extent, suggest that MMP2 may also act as an oncogene in CC development. Additionally, Yi et al. discovered that circRNA–miRNA–mRNA networks in CC pathology may execute vital regulatory functions [32]. This suggests that a direct target gene may be modulated by circ_0051428–miR-885-3p. Therefore, we speculated that the target gene was MMP2. Interestingly, MMP2 is indeed a miR-885-3p downstream target gene. Therefore, we hypothesized that MMP2 may play a role in the regulation of CC progression via miR-885-3p. Unsurprisingly, the deficiency in MMP2 diminished, at least in part, promoting the effects of miR-885-3p downregulation on CC cell invasion and proliferation. Collectively, we believe that silencing circ_0051428 impedes CC development by regulating the miR-885-3p/MMP2 axis.

In conclusion, this study is a basic research from China to uncover the oncogenic role of circ_0051428 in the progression of CC by regulating miR-885-3p/MMP2 axis. These findings highlight a new research direction for the future. However, the lack of analysis of circ_0051428 expression in TCGA data and the correlation between circ_0051428 expression and the prognosis of patients with CC is a limitation of our study and warrants further confirmation in the future.
